# Health Facts Medication Adherence in Transplantation (H-MAT) Study: A Secondary Analysis of Determinants and Outcomes of Medication Nonadherence in Adult Kidney Transplant Recipients

**DOI:** 10.1155/2022/9653847

**Published:** 2022-06-10

**Authors:** Cynthia L. Russell, Heather J. Gotham, An-Lin Cheng, Suman Sahil, Preethi Yerram

**Affiliations:** ^1^School of Nursing and Health Studies, University of Missouri-Kansas City, 2464 Charlotte Street, Kansas City, MO 64108, USA; ^2^Department of Psychiatry & Behavioral Sciences, Stanford University School of Medicine, 1520 Page Mill Road, Palo Alto, CA 94393, USA; ^3^University of Missouri-Kansas City, School of Medicine, Department of Biomedical and Health Informatics, 2411 Holmes Street, Kansas City, MO 64108, USA; ^4^Division of Nephrology, Department of Medicine, University of Missouri-Columbia, 1 Hospital Drive, Columbia, MO 65212, and Nephrology Section, Harry S Truman Veterans Administration Hospital, 800 Hospital Dr, Columbia, MO 65201, USA

## Abstract

**Aims:**

To explore the relationship between determinants and posttransplant medication nonadherence (MNA) in adult kidney transplant recipients, and to examine the relationship between posttransplant MNA and clinical outcomes.

**Methods:**

Using the World Health Organization's model, this retrospective, multicenter, correlational study examined the relationship between determinants, posttransplant MNA, and clinical outcomes in 16,671 adult kidney transplant recipients from the Cerner *Health Facts* national data warehouse.

**Results:**

With 12% MNA, those who were nonadherent were more likely to have the social/economic factors of being younger, single, Caucasian versus Hispanic race, have the condition-related factor of mental health/substance use disorder, and have the healthcare system-related factor of government/health maintenance organization/managed care insurance (*p*′*s* < 0.05). Bivariate correlations indicated both age (OR = 1.006, *p*=0.01) and mental health or substance use disorder diagnosis (OR = 1.26, *p*=0.04) were significant predictors of MNA. Patients were 0.6% more likely to be medication adherent for each year they increased in age and 26% more likely to be MNA if they were diagnosed with a mental health/substance use disorder. Nonadherent patients were less likely to be readmitted, but more likely to have complications after transplant and medication side effects (*p*′*s* < 0.01).

**Conclusions:**

Using one of the largest samples of adult kidney transplant patients, our findings support the WHO model and move the body of medication adherence intervention research forward by clarifying the importance of focusing interventions not only on the patient but on multilevel determinants. Consistent with previous studies, MNA negatively impacts transplant outcomes.

## 1. Introduction

Medication nonadherence (MNA) in adult kidney transplant recipients is estimated to be 36 cases per 100 persons per year [1] and results in a 6- to 7-fold increase in transplant graft failure, hospitalizations, nursing home admissions, and premature deaths [2]. Drug-related morbidity and mortality resulting from nonoptimized medication therapy was $528.4 billion, or 16% of the total United States' healthcare expenditures in 2016 [3]. MNA in transplant costs $33,000 per patient in the first 3 years after transplant [2].

The World Health Organization (WHO) developed a model of five factors that may influence medication adherence: social/economic, patient, condition, therapy, and healthcare team/system factors [4]. Social/economic factors include what we typically think of as social determinants of health. Patient-related factors include patient resources, knowledge, attitudes, beliefs, and perceptions. Condition-related factors include the demands of the patient's illness such as symptom severity, disability level, and disease severity/progression. Therapy-related factors include medical regimen complexity, treatment duration, treatment failures, side effects, and medical support. Healthcare team/system-related factors include such areas as health services/insurance plan reimbursement and provider chronic disease management knowledge/training.

No study has explored multilevel determinants using the WHO model and outcomes of MNA in a large dataset of adult kidney transplant recipients. The current study attempted to address research gaps by using a very large data warehouse of over 34 million patients with 62 million patient encounters, including electronic health record (EHR) and ancillary data sources.

The two purposes of this study were to explore the relationship between determinants and posttransplant MNA in adult kidney transplant recipients and to examine the relationship between posttransplant MNA and clinical outcomes in this same population.

## 2. Materials and Methods

This study is a retrospective, multicenter, correlational analysis of data from *Health Facts*. The Institutional Review Board at the first author's university determined that this study was “nonhuman subjects research” (IRB Protocol ID: 14-567).

### 2.1. Setting

The HIPAA-compliant, deidentified Cerner *Health Facts* data are from the EHR and ancillary data sources from socioeconomically and geographically diverse facilities (e.g., hospital, provider office, specialty clinic, physically distinct site) in which Cerner has a data use agreement. Facilities' participation in *Health Facts* is voluntary, and they individually determine what data elements to add, including billing and encounter data, medication ordering and dispensing, and diagnostic test results. *Health Facts* uses widely adopted coding systems. All diagnoses, medication orders and dispensing, laboratory orders, and specimens are date and time stamped, providing a temporal relationship between treatment patterns and clinical information.

### 2.2. Population

The study sample was gathered from the *Health Facts* proprietary, national data warehouse. The *Health Facts* dataset used in this study was released in December 2016 and covers approximately 11.4 million inpatients, 62 million patient encounters, and 22.9 million emergency patients across 863 facilities.

### 2.3. Sampling

The data from all consecutive patients who were adult (>18 years of age) kidney transplant recipients, with International Classification of Diseases, Ninth Revision (ICD-9) or International Classification of Diseases, Tenth Revision (ICD-10) codes of 55.6 and Z94, respectively (transplant of kidney), were gathered from *Health Facts* between January 2010 and December 2016.

### 2.4. Data Collection

Data from the EHR were obtained from the *Health Facts* data warehouse by the data analyst expert (SS). The EHR is the primary source of phenotypic information specifying social/economic, patient, condition, therapy, and healthcare team/system determinants, posttransplant MNA, and clinical outcomes in adult kidney transplant recipients. The cohort of adult kidney transplant recipients was identified using ICD-9 and 10 codes and/or Current Procedural Terminology (CPT). National Drug Codes (NDC codes), generic names, and brand names were used to identify medications prescribed for adult kidney transplant recipients. No patient-related data were available from the *Health Facts* data warehouse, so this factor was not explored further as a determinant.

The following data definitions were used for the multilevel determinants and outcomes in this study.

#### 2.4.1. Social/Economic Determinants

The social/economic-related determinant data included gender, age, race, and marital status.

#### 2.4.2. Condition-Related Determinant

The condition-related factor data was presence of a diagnosis of a mental health or substance use disorder, ICD-9 290-316 or ICD-10 F01-F99 occurring at any time in the dataset.

#### 2.4.3. Healthcare Team/System-Related Determinant

This determinant was described as payer type including commercial insurance, government insurance (military dependents and other government insurance), HMO/managed care (including Medicaid or Medicare managed care), Medicaid, Medicare, self-pay/free (research), or other insurance (including missing and unknown).

#### 2.4.4. Medication Nonadherence

MNA was defined in the *Health Facts* database for the patient's encounter/visit during which the patient was administered tacrolimus (LOINC 32594.4), cyclosporine (LOINC 4207-7), or mycophenolate mofetil (LOINC 72667-9, 73680-1, 23905-3, 55806-4, 70211-8) as a discharge medication, new medication, or prearrival medication that the patient was not taking, not taking due to complex regimen, not taking due to patient decision, not taking because unable to purchase, or still taking not as prescribed. This included medications administered in the hospital to the patient by providers and/or patient self-reported administration at home but assessed during a clinic visit.

#### 2.4.5. Negative Outcomes

Three negative outcome variables were gleaned from the dataset. Readmission was coded as a readmission to the hospital in the first 3 months after transplant. Complications after kidney transplant were coded if the following ICD or T codes were used: ICD-9 996.81 (complications after kidney transplant), ICD-10 T86.10 (complications after kidney transplant unspecified), T86.11 (kidney transplant rejection), T86.12 (kidney transplant failure), T86.10-T86.19 (other complications after kidney transplant), or T86.1 (complications after kidney transplant). Side effects were coded if the following was used: 995.2, other and unspecified adverse effects of drug, medicinal, and biological substances.

### 2.5. Data Analysis

The data analysis expert (SS) and biostatistician (AC) utilized SAS version 9.4 (SAS Institute, Cary, NC), SQL, and R to perform queries against the *Health Facts* data. Descriptive statistics were computed to characterize the population. Chi-square analyses were conducted to examine relations between MNA and determinants. Bonferroni corrected alpha levels were used for post hoc comparisons conducted for variables with multiple categories. Bivariate correlations such as Spearman correlations or Cramer's V correlations between MNA and potential determinants and outcomes were calculated and examined. To account for the cluster effect of hospital, a logistic regression with random effects was conducted to examine the effect of determinants on MNA. To control for multicollinearity, only significant predictors from binary relationships between the outcome variable and potential predictors were considered and entered into the final model. To examine relations between MNA and outcomes, chi-square analyses were conducted.

### 2.6. Procedure

Key concepts from the theoretical framework were identified in concordance with the study research questions. The *Health Facts* database was then queried to determine which of the theoretical concepts were obtainable from the *Health Facts* database and these queries were then conducted.

## 3. Results

The final sample included 16,671 adult kidney transplant recipients identified across 277 hospitals, including acute care (226) and nonacute (51), teaching (56), nonteaching (137), and unknown teaching status (84). United States' hospitals in the South provided 8,376 patients, with 3,770 patients from the Northeast, 3,202 patients from the West, and 2,517 patients from the Midwest.

Table 1 provides demographics of the sample, as well as delineating the social/economic condition, and healthcare team/system factors by posttransplant medication adherence and nonadherence. No patient-related determinants were gleaned from the *Health Facts* database. Half of adult kidney transplant recipients were female 50.84% (8,474) and a majority were Caucasian 67.26% (10,992). About 10% had a substance use or mental health disorder diagnosis at any time in the dataset, including 31% (510) with a substance use disorder, 27% (451) with a mood disorder (e.g., depression), 26% (423) with an anxiety related disorder, and 16% (262) with another type of mental health disorder. MNA occurred in 12% (1,995) of patients. Compared to adherent patients, nonadherent patients were younger, single, diagnosed with a mental health or substance use disorder, and had government/health maintenance organization (HMO)/managed care insurance. Nonadherent patients were more likely to be Caucasian than Hispanic; there were no significant differences in nonadherence between African Americans, Asian/Pacific Islanders, and Caucasians.

Bivariate correlations showed that most predictors were significantly correlated with nonadherence except gender and race. Marital status, mental health or substance use disorder diagnosis, and age were significant univariate predictors for MNA (all *p*'s <0.01). However, due to multicollinearity (highly correlated predictors which are likely to result in unstable model estimates) and to produce a parsimonious model, only age and mental health or substance use disorder diagnosis were included in a logistic regression with random effects to predict MNA. Both age (OR = 1.006, *p*=0.01) and mental health or substance use disorder diagnosis (OR = 1.26, *p*=0.04) were significant. Patients were 0.6% more likely to be medication adherent for each year they increased in age. Additionally, patients were 26% more likely to be nonadherent if they were diagnosed with a mental health or substance use disorder.

Table 2 shows a comparison of negative patient outcomes by adherence and nonadherence. Nonadherent patients were less likely to be readmitted, but more likely to have complications after transplant and side effects from medications (all *p*'s <0.01).

## 4. Discussion

This study is the first in transplantation using a large dataset and one of the first in chronically ill adults to concurrently explore the association of theory-based social/economic-, condition-, therapy-, and healthcare team/system-related factors with MNA and then examine the relationship between MNA and clinical outcomes. [5–7].

At the bivariate level, social/economic-level determinants of younger age, Caucasian versus Hispanic race, and being single were independent predictors of MNA. In adults, the finding of younger age being associated with MNA in transplant recipients is consistent with the existing literature [8–11]. Likewise, being unmarried has been linked with nonadherence in the transplant literature [12]. However, this is the first study of a large sample to establish Hispanic ethnicity as a protective factor for MNA in transplantation using the WHO model. This is interesting as some studies have found that Hispanic ethnicity is a risk factor for MNA (e.g., diabetes, cardiovascular disease), although there is little recent research in this area [13,14]. Underlying potential causes of the current finding include familism, family support, or a strong belief in God which has been shown to influence medication adherence in kidney transplant recipients [15–18].

For condition-related factors, this study found that the presence of a mental health or substance use disorder diagnosis was related to MNA after transplant. Historically, it has been assumed that mental health and substance use disorders have negative effects on physical health, medical treatment adherence, and transplant outcomes. Current serious mental illness (e.g., psychosis) and substance use disorders are often used as criteria to deny access to transplant in many countries. The research base examining the effects of prior mental health and substance use disorders on transplant outcomes is mixed, with few prospective studies (in part due to the general exclusion of patients with these disorders from transplant), studies across transplant organs, variability in assessment of substance use (use, misuse, and disorder), and inconsistency of when mental illness or substance use is measured (history pretransplant, time of transplant, postoperative, and long-term follow-up) [11,19–21]. While psychotic disorders have frequently been a rule-out for transplant, two studies of patients with serious mental illness, including psychosis, found positive outcomes [21,22], although nonadherence was not specifically examined. Similarly, recent studies examining the effects of a history of substance use disorder on adherence have mixed results [19,23].

For healthcare team/system-related determinants, due to limitations of the *Health Facts* dataset, we were only able to examine the effect of payer type on MNA. Patients using self-pay/free (research) and commercial insurance were less likely to be MNA. This could be due to patients with commercial insurance being more financially well-off and educated. In contrast, patients with government insurance or HMO/managed care plans were more likely than other groups to be MNA. While this is contrary to previous studies of the influence of socialized medicine on MNA, our findings may reflect that government insurance/HMO/managed care in the US did not provide full immunosuppressive medication coverage for patients. In a meta-analysis, Dew et al. found that the immunosuppressive MNA in transplant recipients living in the US was 33%, while it was only 13.5% for those living in Europe and other countries. [1].

These findings have policy implications. With the issuance of the Executive Order on Advancing American Kidney Health by President Trump [24], there is focus and additional resources for promoting the health of those with a transplant so they keep their kidney as long as possible and do not have to rejoin the transplant list. Currently, Medicare Part B only covers kidney transplant recipients' immunosuppressive medications for 36 months after a transplant which can lead to MNA. This study includes the largest sample size to date, documenting the association of immunosuppressive MNA and poor outcomes in adult kidney transplant patients. The outcomes of complications after transplant and side effects from medications were significantly different between adherent and nonadherent patients, consistent with previous findings of a 6- to 7-fold increase in transplant graft failure, hospitalizations, nursing home admissions, and premature deaths with MNA [2].

The 12% MNA rate found in this study is consistent with a prior study [8] but lower than the 36 cases per 100 persons per year found by Dew and colleagues in a meta-analysis [1], the 25% found by Pinsky and colleagues [2], and the 86% found in a study of older kidney transplant patients [25]. This may be due to the hesitancy of healthcare providers to label patients as MNA which could have a negative impact on their ability to rejoin the transplant list if their kidney fails.

In addition, the EHR should more clearly define and document MNA. The taxonomy for defining and describing adherence to medications suggests that adherence to medications is the process by which patients take their medication as prescribed, further divided into three quantifiable phases: “initiation”, “implementation,” and “discontinuation” [26] (p. 691). Providing clear definitions in the EMR would greatly enhance our ability to consistently describe and quantify MNA to aid in the research on MNA. Furthermore, the EMR could be enhanced by including documentation of reasons for MNA.

## 5. Conclusions

In conclusion, our findings support the WHO model and move the body of medication adherence intervention research forward by clarifying the importance of focusing interventions not only on the patient, but on multilevel determinants. Our findings of the influence of multilevel factors on MNA support previous research, while the healthcare system factor provides new evidence of the importance of payer type.

Clearly, more rigorous study is required of the real effect of substance use and mental health disorders on transplant outcomes including MNA. Although other social/economic factors related to nonadherence are not overtly used as reasons to deny transplant, they are signals of health disparities. Studies such as this that identify predictors of MNA after transplant or negative transplant outcomes also alert health care providers to the possible need for more posttransplant services and outreach to assure positive outcomes.

## Figures and Tables

**Table 1:** Condition, therapy, and health care team/system factors by post-transplant medication adherence and nonadherence.

	Overall *n*, % or mean, ±SD	Medication adherent *n*% or mean, ±SD	Medication non-adherent *n*, % or mean, ±SD	Medication non-adherent by category (non-adherence divided by overall)	*p* value
Social/economic determinants					
Gender					
Male	8193 (49.16%)	7184 (48.96%)	1009 (50.58%)	12.32%	0.1765
Female	8474 (50.84%)	7488 (51.04%)	986 (49.42%)	11.64%	

Age in years	53.21 ± 15.97	53.41 ± 16.00	51.7 3± 15.24		<0.0001

Race					
African American	2936 (17.96%)	2585 (17.99%)	351 (17.80%)	11.95%	0.008
Asian/Pacific Islander	2261 (13.83%)	2017 (14.03%)	244 (12.37%)	10.79%	
Caucasian	10992 (67.26%)	9623 (66.97%)	1369 (69.42%)	12.45%^a^	
Hispanic	153 (0.94%)	145 (1.01%)	8 (0.41%)	5.23%^a^	

Marital status					
Divorced	897 (5.83%)	791 (5.85%)	106 (5.64%)	11.82%	<0.0001
Legally separated	160 (1.04%)	152 (1.13%)	8 (0.43%)	5.00%^a^	
Life partner	10 (0.06%)	8 (0.06%)	2 (0.11%)	20.00%	
Married	8142 (52.90%)	7203 (53.32%)	939 (49.95%)	11.53%^b^	
Single	5189 (33.72%)	4460 (33.01%)	729 (38.78%)	14.05%^abc^	
Widowed	992 (6.45%)	896 (6.63%)	96 (5.11%)	9.68%^c^	

Condition-related determinant					
Mental health or substance use diagnosis					
No	15025 (90.13%)	13260 (90.5%)	1765 (88.47%)	11.75%	0.0082
Yes	1646 (9.87%)	1416 (9.65%)	230 (11.53%)	13.97%	

Healthcare team/system-related determinants					
Payer type					
Commercial insurance	2502 (15.01%)	2286 (15.58%)	216 (10.83%)	8.63%^acde^	<0.0001
Government	318 (1.91%)	272 (1.85%)	46 (2.31%)	14.47%^dg^	
HMO/managed care	2713 (16.27%)	2326 (14.85%)	387 (19.40%)	14.26%^cf^	
Medicaid	1321 (7.92%)	1178 (8.03%)	143 (7.17%)	10.82%	
Medicare	6733 (40.39%)	5909 (40.26%)	824 (41.30%)	12.24%^ab^	
Self-pay/free (research)	649 (3.89%)	601 (4.10%)	48 (2.41%)	7.40%^bfgh^	
Others	2435 (14.61%)	2104 (14.34%)	331 (16.59%)	13.59%^eh^	

**Table 2:** Comparison of medication adherent and medication non-adherent patients on negative outcomes.

	Medication adherent *n*, %	Medication nonadherent *n*%	*p* value
Readmission			
No	13697 (93.33%)	1894 (94.94%)	0.0062^*∗*^
Yes	979 (6.67%)	101 (5.06%)	

Complications after transplant			
No	13637 (92.92%)	1736 (87.02%)	<0.0001^*∗*^
Yes	1039 (7.08%)	259 (12.98%)	

Side effects			
No	14540 (99.07%)	1962 (98.35%)	0.0023^*∗*^
Yes	136 (0.93%)	33 (1.65%)	

^*∗*^=*p* < 0.05.

## Data Availability

Data supporting the conclusion in this study may be accessed via the Health Facts database.
